# Longitudinal Trajectories of Multiple Nicotine Product Use Among Youths in the Population Assessment of Tobacco and Health Study

**DOI:** 10.1001/jamanetworkopen.2022.3549

**Published:** 2022-03-23

**Authors:** Patricia Simon, Yannuo Jiang, Eugenia Buta, Carolyn E. Sartor, Suchitra Krishnan-Sarin, Ralitza Gueorguieva

**Affiliations:** 1 Department of Psychiatry, Yale School of Medicine, New Haven, Connecticut; 2 Department of Biostatistics, Yale School of Public Health, New Haven, Connecticut

## Abstract

**Importance:**

Multiple nicotine product use (MNPU) among youths is a significant public health concern. Much remains unknown about the patterns of MNPU in youths, including how socioecological factors influence trajectories of MNPU, which may inform targeted prevention.

**Objective:**

To identify longitudinal trajectories of MNPU and characterize them according to socioecological factors associated with tobacco use.

**Design, Setting, and Participants:**

This US-based longitudinal survey study used data from waves 1 (September 12, 2013, to December 14, 2014) through 4 (December 1, 2016, to January 3, 2018) of the Population Assessment of Tobacco and Health (PATH) study. Participants included 10 086 youths (aged 12-17 years) at wave 1, with follow-up data at waves 2 to 4 (assessed approximately 1 year apart) in the youth or adult data sets. Data were analyzed from January 15, 2020, to December 22, 2021.

**Exposures:**

Socioecological factors at wave 1.

**Main Outcomes and Measures:**

Outcome variables were days of use in the past 30 days of 4 products: cigarettes, e-cigarettes, cigars, and smokeless tobacco. Factors associated with use of the nicotine products that were collected at wave 1 included sociodemographic factors, internalizing and externalizing symptoms, living with a tobacco user, rules about tobacco use at home, conversations with parents about not using tobacco, tobacco accessibility, and exposure to advertising. Multitrajectory latent class growth analysis was used to identify distinct subgroups with similar patterns of use over time. Multinomial logistic regression models were used to investigate factors associated with class membership. Weights were applied to all data except frequencies to account for the complex survey design.

**Results:**

Of the 10 086 youths included in the analysis, 5142 (51.2%) self-identified as male; 4792 (54.7%) were non-Hispanic White; and 5315 (50.6%) were aged 12 to 14 years. Six latent trajectory classes were identified: nonuse (8056 [78.2%]), experimentation (908 [9.8%]), increasing e-cigarette/cigarette use (359 [4.0%]), increasing cigarette/cigar use (320 [3.3%]), decreasing cigarette/e-cigarette/cigar use (302 [3.2%]), and stable smokeless tobacco/cigarette use (141 [1.6%]). Compared with the nonuse class, being older (odds ratio [OR] range, 2.54 [95% CI, 1.94-3.32] to 9.49 [95% CI, 6.03-14.93]), being female (OR range, 0.06 [95% CI, 0.03-0.14] to 0.71 [95% CI, 0.53-0.94]), living with a tobacco user (OR range, 1.43 [95% CI, 1.11-1.83] to 4.94 [95% CI, 3.43-7.13]), and having relaxed rules about noncombustible tobacco product use at home (OR range, 1.41 [95% CI, 1.02-1.94] to 3.42 [95% CI, 1.74-6.75]) were associated with classification in all the use classes. A high degree of difficulty accessing tobacco was associated with lower odds of membership in the increasing cigarette/cigar use vs nonuse classes (OR, 0.62 [95% CI, 0.40-0.98]).

**Conclusions and Relevance:**

These survey results highlight the heterogeneity of longitudinal pathways of MNPU in US youths and suggest directions for future prevention and regulatory efforts directed at tobacco use behaviors in this population.

## Key Points

**Question:**

What are the trajectories of multiple nicotine product use among US youths?

**Findings:**

In this 4-wave, nationally representative survey study that included 10 086 youths, 6 trajectories with weighted proportions emerged: nonuse (8056 [78.2%]), experimentation (908 [9.8%]), increasing e-cigarette/cigarette use (359 [4.0%]), increasing cigarette/cigar use (320 [3.3%]), decreasing cigarette/e-cigarette/cigar use (302 [3.2%]), and stable smokeless tobacco/cigarette use (141 [1.6%]).

**Meaning:**

These results highlight the heterogeneity of longitudinal pathways of multiple nicotine product use among youths in the US and suggest directions for future prevention and regulatory efforts directed at nicotine product use behaviors in this population.

## Teaser

This survey study develops a comprehensive model of the association of a range of intrapersonal, family, and environmental factors with longitudinal patterns of multiple nicotine product use by US adolescents in a trajectory analysis framework.

## Introduction

Recent evidence suggests that use of tobacco products by youths has decreased from 6.2 million in 2019 to 4.5 million in 2020.^1^ Despite this downward trend, in 2020, nearly 1 in 4 US high school students and approximately 1 in 15 middle school students reported past 30-day use of a tobacco product.^1^ Moreover, 34.7% of youth tobacco users use multiple tobacco products,^1^ which is associated with an increased risk for nicotine use disorder.^2^ Understanding how pathways of multiple nicotine product use (MNPU) vary among youths is critical for the development of prevention interventions and should therefore be a major public health goal.

Capturing longitudinal heterogeneity is challenging because of the possibility of multiple patterns. Analysis of a single wave of MNPU data from a nationally representative sample of youths yielded 116 cross-sectional combinations.^3^ Analyzing all possible patterns across multiple waves is unwieldy, with data sparseness and subjective decisions about product groupings potentially leading to unreproducible and ultimately uninformative results. In contrast to observed pattern approaches, latent variable approaches allow the “big picture” to emerge by jointly leveraging formal statistical criteria and conceptual considerations to identify the most parsimonious, meaningful patterns and trends and facilitate identification of factors associated with these patterns.^4,5^ Latent transition analysis has identified unobservable (latent) subgroups based on responses to multiple product use questions and evaluated movement between these subgroups over time but has been limited to examining transitions across 2 waves of data.^6,7,8^ A recent investigation modeled past 30-day use (vs no use) data for 3 product groupings (e-cigarette, cigarette, and other tobacco) across multiple waves of data and identified 5 latent trajectories of MNPU.^9^

The present study was designed to address key gaps in the literature. First, we considered more products and included cigar and smokeless tobacco products as separate categories because a substantial proportion of youths^1^ and different demographic groups^10^ are attracted to them. Second, we assessed the number of days of nicotine product use in the past 30 days, which allows for the differentiation of patterns of experimentation vs more consistent use. Third, we sought to identify distinctions among MNPU patterns with respect to socioecological factors to inform targeted interventions. In contrast to the well-established associations with cigarette smoking,^11^ our understanding of the role of socioecological factors in MNPU is nascent. Demographic factors such as male sex, lower parental educational levels, and being older have been linked to a greater likelihood of transition to MNPU,^7^ as have mental health problems, including depression, anxiety, and attention-deficit/hyperactivity disorder.^12^ However, other factors associated with decreased risk of cigarette smoking have yet to be investigated in relation to MNPU, including less accessibility of tobacco products, less exposure to tobacco advertising,^13,14,15^ fewer tobacco users in the youth’s environment,^16^ parent communication about not smoking,^17,18,19^ and home smoking bans.^19^ The goal of this study was to develop a more comprehensive model of the association of a range of intrapersonal, family, and environmental factors with longitudinal patterns of MNPU in a trajectory analysis framework.

## Methods

### Participants and Procedures

The data for this survey study were drawn from waves 1 to 4 of the Population Assessment of Tobacco and Health (PATH) study. Participants were aged 12 to 17 years at wave 1 (n = 13 651). Data were collected annually (wave 1, September 12, 2013, to December 14, 2014; wave 2, October 23, 2014, to October 30, 2015; wave 3, October 19, 2015, to October 23, 2016; and wave 4, December 1, 2016, to January 3, 2018). We performed analyses on the subsample (10 086 of 13 651 [73.9%]) who had longitudinal weights, including those 18 years or older at follow-up (part of the adult data set). Consistent with the American Association for Public Opinion Research (AAPOR) reporting guidelines, details on recruitment and design of the PATH study can be found in prior publications^20^ and at the National Addiction & HIV Data Archive Program website.^21^ Briefly, the recruitment strategy consisted of a stratified address-based, area-probability sampling design at wave 1, with participants selected via an in-person household screener. Following informed consent procedures, data were collected using audio-computer–assisted self-interviews administered in English or Spanish. The PATH study was conducted by Westat and approved by the Westat institutional review board. The Yale University Institutional Review Board approved the secondary data analyses.

### Measures

#### Use of Tobacco Products

Outcome variables were collected at waves 1 through 4. Survey participants were asked to indicate the number of days in the past 30 days in which they used each of the following tobacco products: cigarettes, e-cigarettes, traditional cigars, cigarillos, filtered cigars, and smokeless tobacco. The answers for traditional cigars, cigarillos, and filtered cigars were combined into a single product (cigar) by taking their maximum so that all products could be rated on the same scale of past 30-day use in our model.

#### Socioecological Factors

All socioecological variables were collected at wave 1. These included intrapersonal factors (sex, age, parental educational level, race and ethnicity, externalizing symptoms,^22^ and internalizing symptoms^22^), family factors (living with a tobacco user, rules about the use of combustible and noncombustible tobacco products in the home, and conversations with parents about tobacco use), and environmental factors (US Census region, tobacco accessibility, and exposure to advertising) (Table 1).

**Table 1.** Measurement of Intrapersonal, Family, and Environmental Factors Assessed

Factor	Description
**Intrapersonal**	
Age^a^	12-14 vs 15-17 y. To protect participant privacy, the public use files only provide dichotomized age, with the 2 age ranges reflecting early and late adolescence, respectively.
Parental educational level^b^	More than high school vs less than high school or completed high school
Race and ethnicity^a^^,^^b^	Hispanic, non-Hispanic Black, non-Hispanic White, and other. Regarding the category “other,” although the PATH survey asked participants about several categories (American Indian or Alaska Native, Asian Indian, Chinese, Filipino, Guamanian or Chamorro, Japanese, Korean, Native Hawaiian, Samoan, Vietnamese, other Asian, and other Pacific Islander), given the limited information available in the public use files, it was not possible to determine whether all the racial and ethnic groups probed are reflected in our subset of the data.
Sex	Female vs male
Externalizing symptoms	The items come from the GAIN-SS.^22^ Participants were asked when they last did any of the following things ≥2 times: (1) lied or conned to get things you wanted or to avoid having to do something; (2) had a hard time paying attention at school, work, or home; (3) had a hard time listening to instructions at school, work, or home; (4) were a bully or threatened other people; (5) started physical fights with other people; (6) felt restless or the need to run around or climb on things; and (7) gave answers before the other person finished asking the question (1 indicates past month; 2, 2-12 mo ago; 3, >1 y ago; and 4, never). Each item was converted to a binary response (1 indicates behavior occurred in past 12 mo; 0, otherwise) before they were summed to create a scale from 0 to 7.
Internalizing symptoms	The items come from the GAIN-SS.^22^ Participants were asked when they last had significant problems with (1) feeling very trapped, lonely, sad, blue, depressed, or hopeless about the future; (2) sleep trouble, such as bad dreams, sleeping restlessly, or falling asleep during the day; (3) feeling very anxious, nervous, tense, scared, panicked, or as if something bad was going to happen; and (4) becoming very distressed and upset when something reminded you of the past (1 indicates past month; 2, 2-12 mo ago; 3, >1 y ago; and 4, never). Each item was converted to a binary response (1 indicates behavior occurred in past 12 mo; 0, otherwise) before they were summed to create a scale from 0 to 4.
**Familial**	
Conversations about not using tobacco	Participants reported whether their parents or guardians talked with them in the past 12 mo about not using any type of tobacco product (1 indicates yes; 0, no).
Other person in home uses tobacco	Participants reported whether anyone who lives with them currently (1) uses cigarettes, cigars, cigarillos, or filtered cigars; (2) uses smokeless or other tobacco only; and (3) no one living in the home uses tobacco. Categories 1 and 2 were combined into a single “yes” category.
Rules about using combustible tobacco at home	Participants were asked which statement best describes the rules about smoking a tobacco product inside their home for tobacco products that are burned, such as cigarettes, cigars, pipes, or hookah, where 1 indicates not allowed anywhere or any time; 2, allowed in some places or sometimes; and 3, allowed anywhere or any time.
Rules about using noncombustible tobacco at home	Participants were asked which statement best describes the rules about smoking a tobacco product inside their home for tobacco products that are not burned, such as smokeless tobacco, dissolvable tobacco, and electronic cigarettes, where 1 indicates not allowed anywhere or any time; 2, allowed in some places or sometimes; and 3, allowed anywhere or any time.
**Environmental**	
US Census region	Northeast, Midwest, South, or West
Exposure to advertising	Participants were shown 20 advertisements promoting the use of tobacco products and were asked: “In the past 12 months, have you seen this advertisement before this study? (yes or no).” We created 1 overall binary variable where 1 indicates seen any advertisements; 0, not seen advertisements.
Tobacco accessibility	Participants reported how easy they think it is for people their age to buy tobacco products in a store, where 1 indicates very easy; 2, somewhat easy; 3, somewhat difficult; and 4, very difficult.

Abbreviations: GAIN-SS, Global Appraisal of Individual Needs Short Screener; PATH, Population Assessment of Tobacco and Health.

^a^ For missing data, imputed values available with the PATH data were used.

^b^ Owing to low sample sizes in some cells, parental educational level was reduced to 2 categories.

### Statistical Analysis

Data were analyzed from January 15, 2020, to December 22, 2021. Latent class growth analysis was applied using PROC TRAJ in SAS, version 9.4 (SAS Institute Inc) to identify distinct latent groups that were similar with respect to simultaneously modeled cigarette, e-cigarette, cigar, and smokeless tobacco use over time. We used a Poisson distribution for the outcomes and a quadratic polynomial for the trajectories. We fit latent class growth analysis without covariates and selected the number of classes based on the lowest bayesian information criterion. We also computed the mean posterior probabilities of assignment in each class, with values higher than 0.7 considered acceptable.^5^ Classes were named based on the most prominent class characteristics. We fitted a multivariable multinomial logistic regression model using PROC SURVEY LOGISTIC in SAS, version 9.4, to investigate factors associated with class membership, with each individual assigned to the class for which they have the highest posterior probability. This model included complete cases only, which was appropriate because the amount of missing data was low (12% had missing data on ≥1 of the variables). All results are weighted (except frequencies) and take into consideration the complex design of the PATH survey.

## Results

Of the 10 086 participants included in the analysis at wave 1, 5315 (50.6%) were 12 to 14 years of age and 4771 (49.4%) were 15 to 17 years of age. A total of 5142 participants (51.2%) self-identified as male and 4944 (48.8%) as female. In terms of race and ethnicity data, which are important for evaluating well-established disparities in tobacco use, 2935 participants (22.2%) were Hispanic, 1430 (13.9%) were non-Hispanic Black, 4792 (54.7%) were non-Hispanic White, and 929 (9.2%) were other race or ethnicity (categories include American Indian or Alaska Native, Asian Indian, Chinese, Filipino, Guamanian or Chamorro, Japanese, Korean, Native Hawaiian, Samoan, Vietnamese, other Asian, and other Pacific Islander). Regarding the category “other,” we could not determine whether all the racial and ethnic groups listed are reflected in our subset of the data owing to the limited information available in the public use files. Almost two-thirds (6040 [64.2%]) had a parent with more than a high school education. Detailed participant characteristics are provided in Table 2.

**Table 2.** Participant Characteristics at Wave 1 Overall and by Latent Class^a^

Characteristic	All participants (N = 10 086)	Use of nicotine product					
		Class 1: nonuse (n = 8056)	Class 2: increasing cigarette/cigar (n = 320)	Class 3: experimentation (n = 908)	Class 4: increasing e-cigarette/cigarette (n = 359)	Class 5: stable smokeless tobacco/cigarette (n = 141)	Class 6: decreasing cigarette/e-cigarette/cigar (n = 302)
**Intrapersonal**							
Age, y							
12-14	5315 (50.6)	4731 (56.8)	88 (26.1)	285 (30.9)	127 (33.5)	41 (26.9)	43 (13.3)
15-17	4771 (49.4)	3325 (43.2)	232 (73.9)	623 (69.1)	232 (66.5)	100 (73.1)	259 (86.7)
Parental educational level							
High school or less	3976 (35.8)	3141 (35.1)	148 (42.2)	341 (34.1)	135 (35.2)	58 (40.4)	153 (48.4)
More than high school	6040 (64.2)	4858 (64.9)	169 (57.8)	558 (65.9)	224 (64.8)	82 (59.6)	149 (51.6)
Race and ethnicity							
Hispanic	2935 (22.2)	2463 (23.3)	80 (20)	241 (20.7)	81 (17.8)	18 (9)	52 (14.1)
Non-Hispanic Black	1430 (13.9)	1210 (14.9)	53 (14.8)	123 (13.2)	16 (4)	4 (2.3)	24 (8.2)
Non-Hispanic White	4792 (54.7)	3643 (52.1)	159 (58.3)	460 (57.8)	225 (69.8)	112 (84.4)	193 (71)
Other^b^	929 (9.2)	740 (9.7)	28 (7)	84 (8.3)	37 (8.3)	7 (4.3)	33 (6.8)
Sex							
Male	5142 (51.2)	3962 (49.2)	170 (52.4)	490 (54.4)	232 (64.7)	132 (93.9)	156 (51.8)
Female	4944 (48.8)	4094 (50.8)	150 (47.6)	418 (45.6)	127 (35.3)	9 (6.1)	146 (48.2)
No. of externalizing symptoms, mean (SD)	2.56 (1.92)	2.37 (1.87)	3.11 (1.97)	3.18 (1.91)	3.43 (1.91)	2.87 (2.18)	3.38 (2.06)
No. of internalizing symptoms, mean (SD)	1.85 (1.56)	1.73 (1.53)	2.32 (1.59)	2.25 (1.58)	2.39 (1.58)	1.61 (1.58)	2.50 (1.57)
**Familial**							
Conversations about not using tobacco							
Yes	5098 (50.8)	3980 (49.6)	175 (53.6)	489 (53.7)	195 (54.1)	89 (63.2)	170 (56.7)
No	4900 (49.2)	3996 (50.4)	145 (46.4)	415 (46.3)	162 (45.9)	51 (36.8)	131 (43.3)
Other person in home uses tobacco							
No	6582 (66.5)	5605 (71.2)	136 (44.4)	501 (56.2)	199 (57.2)	60 (45.6)	81 (26.8)
Yes	3423 (33.5)	2384 (28.8)	181 (55.6)	397 (43.8)	160 (42.8)	80 (54.4)	221 (73.2)
Rules about using combustible tobacco at home							
It is not allowed anywhere or at any time inside my home	8125 (81.7)	6684 (84.5)	214 (68.2)	682 (75.2)	274 (76.8)	100 (71.1)	171 (59.7)
It is allowed in some places or at some times inside my home	1174 (11.6)	836 (10.3)	70 (21.8)	129 (14.1)	49 (13.9)	23 (16)	67 (21)
It is allowed anywhere and at any time inside my home	673 (6.6)	435 (5.2)	32 (9.9)	92 (10.7)	34 (9.3)	18 (12.9)	62 (19.3)
Rules about using noncombustible tobacco at home							
It is not allowed anywhere or at any time inside my home	8011 (80.7)	6659 (84.3)	210 (67.7)	663 (72.8)	246 (69.2)	83 (58.7)	150 (52.5)
It is allowed in some places or at some times inside my home	1016 (10.4)	701 (8.9)	56 (16.9)	120 (14.1)	55 (15.4)	23 (15.7)	61 (19.3)
It is allowed anywhere and at any time inside my home	857 (9)	527 (6.7)	46 (15.4)	112 (13.1)	52 (15.4)	34 (25.6)	86 (28.2)
**Environmental**							
US Census region							
Northeast	1440 (16.8)	1130 (16.6)	46 (16.5)	137 (17.7)	59 (18.7)	19 (16.5)	49 (18)
Midwest	2260 (21.6)	1716 (20.7)	86 (25.5)	226 (23.7)	104 (26.7)	43 (26.1)	85 (25.2)
South	3765 (37.6)	3054 (37.9)	121 (37.8)	328 (36.9)	97 (29.8)	57 (43.3)	108 (37.5)
West	2621 (23.9)	2156 (24.7)	67 (20.2)	217 (21.7)	99 (24.8)	22 (14.1)	60 (19.3)
Exposure to tobacco advertising							
No	5214 (53.8)	4415 (57.2)	139 (45.3)	354 (41.4)	146 (42)	40 (29)	120 (41.4)
Yes	4592 (46.2)	3411 (42.8)	173 (54.7)	529 (58.6)	208 (58)	95 (71)	176 (58.6)
Tobacco accessibility							
Very easy	1080 (11.2)	783 (10.2)	49 (17.3)	130 (14.6)	49 (13.5)	25 (18.5)	44 (14.6)
Somewhat easy	2525 (26.5)	1938 (25.4)	98 (32.5)	277 (32.2)	102 (31.4)	29 (20.6)	81 (26.4)
Somewhat difficult	2983 (30.4)	2397 (30.8)	95 (28.9)	270 (29.2)	92 (26)	42 (30.3)	87 (31.1)
Very difficult	3284 (31.9)	2748 (33.6)	71 (21.3)	223 (24)	109 (29)	45 (30.6)	88 (27.9)

^a^ Unless otherwise indicated, data are expressed as unweighted number (weighted percentage) of patients. Means (SD) are weighted.

^b^ Includes American Indian or Alaska Native, Asian Indian, Chinese, Filipino, Guamanian or Chamorro, Japanese, Korean, Native Hawaiian, Samoan, Vietnamese, other Asian, and other Pacific Islander. Note that it was not possible to determine whether all these racial and ethnic groups are reflected in our subset of the data owing to the limited information available in public use files.

The 6-class latent class growth analysis model was chosen as our final model because it had the maximum number of classes that converged and minimized the bayesian information criteria (Table 3). The mean posterior probabilities of class membership were at least 0.95 (well above the threshold of 0.70 [Table 3]) for all 6 classes, indicating very good model confidence in assigning participants to classes. We describe the 6 classes (Figure) as follows:

- class 1: nonuse (8056 [78.2%]), characterized as consistent low frequency of use across products;
- class 2: increasing cigarette/cigar use (320 [3.3%]), characterized as increasing mean (SD) cigarette use from 0.21 (0.79) days at wave 1 to 12.10 (11.01) days at wave 4 and cigar use from 0.36 (2.02) to 4.77 (8.96) days, respectively (eTable in the Supplement for observed means);
- class 3: experimentation (908 [9.8%]), characterized as less than 1 day of use of almost all products across all waves (the only exception being a mean [SD] of 1.22 [2.28] days of e-cigarette use at wave 4);
- class 4: increasing e-cigarette/cigarette use (359 [4.0%]), characterized as increasing mean (SD) e-cigarette use (0.97 [4.10] days at wave 1 to 11.27 [11.00] days at wave 4) and cigarette use (0.17 [0.63] days at wave 1 to 2.70 [5.26] days at wave 4);
- class 5: stable smokeless tobacco/cigarette use (141 [1.6%]), characterized as consistent mean (SD) days of smokeless tobacco use (10.33 [12.96] at wave 1 to 13.58 [12.08] at wave 3) and cigarette use (range, 2.06 [5.08] at wave 2 to 4.09 [7.23] at wave 4); and
- class 6: decreasing cigarette/e-cigarette/cigar use (302 [3.2%]), characterized as decreasing mean (SD) days of use of cigarettes (14.94 [12.24] to 10.26 [11.43]), e-cigarettes (2.94 [7.40] to 1.07 [3.39]), and cigars (2.68 [5.92] to 0.88 [3.45] days) from wave 1 to wave 4, respectively.

In sum, youths who used tobacco typically had a primary product they used for at least 10 days per month and a secondary product they used 2 to 5 days per month during at least 1 wave. It is noteworthy that 1 class (decreasing cigarette/e-cigarette/cigar use) included 2 secondary products. In addition, 2 of the trajectories represented youth who had already initiated use at wave 1. In the decreasing cigarette/e-cigarette/cigar trajectory, participants were reporting cigarette use approximately 15 days per month at wave 1. In the stable smokeless tobacco/cigarette trajectory, participants were reporting smokeless tobacco use approximately 10 days per month at wave 1.

**Table 3.** Model Fit Statistics

Class	Log-likelihood	BIC	AIC	Mean posterior probabilities in each class (increasing order)
1	–211 774.1332	423 658.9	423 572.3	1
2	−121 182.6439	242 595.8	242 415.3	.99; .999
3	−105 161.2973	210 672.9	210 398.6	.99; .99; .998
4	−98 723.86447	197 917.9	197 549.7	.98; .99; .995; .999
5	−89 219.12875	179 028.2	178 566.3	.96; .98; .996; .99; .999
6	−85 156.20121	171 022.2	170 466.4	.95; .97; .98; .99; .99; .997
7	Did not converge	NA	NA	NA

Abbreviations: AIC, Akaike information criteria; BIC, bayesian information criteria; NA, not applicable.

**Figure.**
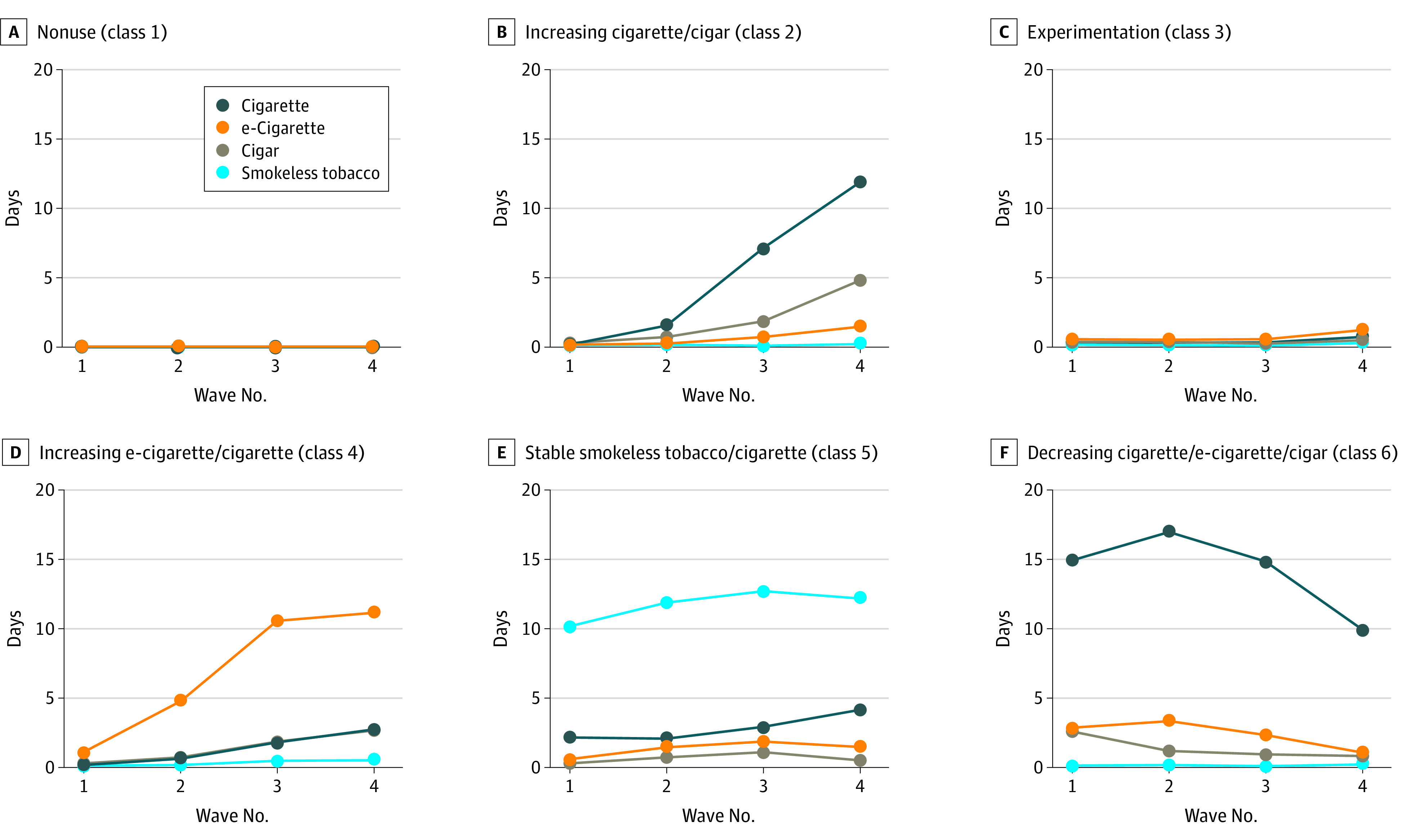
Joint Trajectories of Cigarette, e-Cigarette, Cigar, and Smokeless Tobacco Use During Waves 1 to 4 of Population Assessment of Tobacco and Health Youth Data

In the fully adjusted model with nonuse as the reference class, participants aged 15 to 17 years (odds ratio [OR] range, 2.54 [95% CI, 1.94-3.32] to 9.49 [95% CI, 6.03-14.93]) were more likely to belong to the 5 classes that used nicotine products (Table 4). Girls were less likely than boys to belong to the classes that used nicotine products (OR range, 0.06 [95% CI, 0.03-0.14] to 0.71 [95% CI, 0.53-0.94]). Compared with the other racial and ethnic groups, non-Hispanic Black and other participants were more likely to belong to classes 4 through 6 (OR range, 0.11 [95% CI, 0.04-0.30] to 0.43 [95% CI, 0.29-0.65]). Participants who were exposed to tobacco advertising were more likely to belong to classes 3 through 6 than their nonexposed counterparts (OR range, 1.37 [95% CI, 1.04-1.82] to 2.78 [95% CI, 1.79-4.32]). Higher externalizing symptoms were associated with increased odds of belonging to all the classes that used products except stable smokeless tobacco/cigarette use (OR range, 1.11 [95% CI, 0.97-1.26] to 1.19 [95% CI, 1.09-1.29]). Another person in the home using tobacco (OR range, 1.43 [95% CI, 1.11-1.83] to 4.94 [95% CI, 3.43-7.13]) and absence of restrictions on noncombustible tobacco use (anywhere at any time) inside the home (OR range, 1.41 [95% CI, 1.02-1.94] to 3.42 [95% CI, 1.74-6.75]) were associated with higher odds of belonging to the 5 nicotine product–using classes. Compared with the nonuse class, absence of restrictions on combustible tobacco use inside the home was associated with higher odds of belonging to the experimentation class (OR, 1.58 [95% CI, 1.10-2.29]) and decreasing use of cigarette/e-cigarette/cigar class (OR, 1.70 [95% CI, 1.05-2.75]). Having a conversation with parents about using tobacco was associated with a higher chance of being in classes 3 (OR, 1.19 [95% CI, 1.01-1.40]), 5 (OR, 1.84 [95% CI, 1.15-2.95]), and 6 (OR, 1.51 [95% CI, 1.12-2.02]) than the nonusing class.

**Table 4.** AORs for Belonging to Each Class Compared With the Nonuse Class (Class 1)

Characteristic^a^	Use of nicotine product									
	Class 2: increasing cigarette/cigar		Class 3: experimentation		Class 4: increasing e-cigarette/cigarette		Class 5: stable smokeless tobacco/cigarette		Class 6: decreasing cigarette/e-cigarette/cigar	
	AOR (95% CI)	*P* value	AOR (95% CI)	*P* value	AOR (95% CI)	*P* value	AOR (95% CI)	*P* value	AOR (95% CI)	*P* value
**Intrapersonal**										
Aged 15 to 17 y	3.64 (2.68-4.96)	<.001	2.68 (2.24-3.21)	<.001	2.54 (1.94-3.32)	<.001	4.30 (2.81-6.57)	<.001	9.49 (6.03-14.93)	<.001
Parental educational level more than high school	0.77 (0.58-1.02)	.07	1.08 (0.90-1.31)	.41	0.91 (0.69-1.21)	.53	0.72 (0.44-1.19)	.19	0.62 (0.49-0.78)	<.001
Race and ethnicity										
Hispanic	0.88 (0.64-1.21)	.43	0.89 (0.72-1.11)	.29	0.66 (0.44-0.98)	.04	0.29 (0.14-0.57)	<.001	0.53 (0.36-0.77)	.001
Non-Hispanic Black or other^b^	0.77 (0.56-1.05)	.10	0.80 (0.63-1.00)	.05	0.40 (0.28-0.57)	<.001	0.11 (0.04-0.30)	<.001	0.43 (0.29-0.65)	<.001
Girls	0.69 (0.53-0.89)	.004	0.69 (0.58-0.81)	<.001	0.43 (0.33-0.56)	<.001	0.06 (0.03-0.14)	<.001	0.71 (0.53-0.94)	.02
Externalizing symptoms (0-7)	1.11 (1.02-1.21)	.02	1.14 (1.08-1.20)	<.001	1.19 (1.09-1.29)	<.001	1.11 (0.97-1.26)	.12	1.17 (1.05-1.30)	.005
Internalizing symptoms (0-4)	1.10 (1.00-1.22)	.05	1.09 (1.03-1.16)	.004	1.18 (1.08-1.30)	<.001	0.94 (0.81-1.09)	.41	1.17 (1.06-1.30)	.003
**Familial**										
Conversations about not using tobacco	1.26 (0.94-1.69)	.12	1.19 (1.01-1.40)	.03	1.14 (0.86-1.51)	.35	1.84 (1.15-2.95)	.01	1.51 (1.12-2.02)	.007
Other person in home uses tobacco	2.51 (1.90-3.31)	<.001	1.57 (1.30-1.90)	<.001	1.43 (1.11-1.83)	.006	2.23 (1.38-3.59)	.001	4.94 (3.43-7.13)	<.001
Rules about using combustible tobacco at home										
It is allowed in some places or at some times inside my home	1.54 (1.08-2.18)	.02	1.04 (0.78-1.39)	.77	0.94 (0.62-1.44)	.78	1.06 (0.54-2.07)	.86	1.10 (0.70-1.73)	.66
It is allowed anywhere and at any time inside my home	1.43 (0.91-2.25)	.12	1.58 (1.10-2.29)	.01	1.13 (0.69-1.86)	.62	1.07 (0.52-2.19)	.86	1.70 (1.05-2.75)	.03
Rules about using noncombustible tobacco at home										
It is allowed in some places or at some times inside my home	1.16 (0.75-1.79)	.50	1.32 (1.01-1.73)	.04	1.57 (1.08-2.29)	.02	1.30 (0.71-2.38)	.40	1.60 (1.04-2.44)	.03
It is allowed anywhere and at any time inside my home	1.57 (1.03-2.39)	.04	1.41 (1.02-1.94)	.04	2.04 (1.35-3.08)	<.001	3.42 (1.74-6.75)	<.001	2.19 (1.39-3.44)	<.001
**Environmental**										
US Census region										
Midwest	1.20 (0.76-1.89)	.43	1.02 (0.76-1.37)	.89	1.12 (0.72-1.74)	.63	0.97 (0.46-2.04)	.93	0.92 (0.58-1.45)	.70
South	0.99 (0.64-1.52)	.95	0.93 (0.71-1.20)	.57	0.81 (0.51-1.28)	.36	1.16 (0.72-1.87)	.54	0.82 (0.57-1.19)	.30
West	0.97 (0.59-1.60)	.91	0.89 (0.66-1.20)	.44	0.99 (0.68-1.44)	.96	0.78 (0.42-1.45)	.43	0.81 (0.52-1.26)	.35
Exposure to tobacco advertising	1.22 (0.93-1.61)	.15	1.55 (1.30-1.86)	<.001	1.54 (1.19-1.99)	.001	2.78 (1.79-4.32)	<.001	1.37 (1.04-1.82)	.03
Tobacco accessibility										
Somewhat easy	0.85 (0.60-1.02)	.34	0.98 (0.76-1.26)	.85	0.94 (0.65-1.36)	.74	0.48 (0.26-0.91)	.02	0.76 (0.48-1.19)	.22
Somewhat difficult	0.69 (0.45-1.06)	.09	0.78 (0.61-1.01)	.05	0.71 (0.48-1.06)	.09	0.52 (0.27-1.02)	.06	0.88 (0.60-1.29)	.51
Very difficult	0.62 (0.40-0.98)	.04	0.80 (0.61-1.06)	.12	0.93 (0.60-1.44)	.73	0.60 (0.31-1.17)	.13	0.92 (0.58-1.46)	.72

Abbreviation: AOR, adjusted odds ratio.

^a^ Reference groups include 12 to 14 years for age, high school or less for parental educational level, non-Hispanic White for race and ethnicity, boys for sex, no conversations about tobacco use, no use of combustible or noncombustible tobacco allowed at home, Northeast Census region, no exposure to advertising, and very easy accessibility of tobacco.

^b^ Includes American Indian or Alaska Native, Asian Indian, Chinese, Filipino, Guamanian or Chamorro, Japanese, Korean, Native Hawaiian, Samoan, Vietnamese, other Asian, and other Pacific Islander. Note that it was not possible to determine whether all these racial and ethnic groups are reflected in our subset of the data owing to the limited information available in public use files.

## Discussion

This survey study identified distinct trajectories of MNPU among US youths and socioecologically important variables associated with trajectory membership using waves 1 to 4 of the nationally representative PATH data set. To our knowledge, this is the first study to characterize multiple latent trajectories of youth MNPU by type of tobacco product used while examining how the number of days of use fluctuates over time. We observed that trajectories for youths with MNPU typically had a primary product that was used for at least 10 days per month and a secondary product that was used 2 to 5 days per month during at least 1 year of a 4-year period. To date, no prior studies have included enough specificity regarding the frequency of use to make such a determination.

Although thresholds for problematic levels of use among youths are not well established, the mean days of use for the primary (10 d/mo) and secondary (2-5 d/mo) products may be considered relatively high and relatively moderate, respectively.^23,24^ Further, although the days of use were less than half of the month, these levels of use are concerning because nicotine dependence symptoms may occur in adolescents who smoke only a few cigarettes.^25^ Moreover, occasional cigarette use has been identified as a strong predictor of progression to daily use, lower cessation rates, and higher relapse rates.^26^

Regarding the sequencing of use, 2 of the trajectories (increasing cigarette/cigar and increasing e-cigarette/cigarette use) represented youths who steadily escalated use of the primary product, with use of the secondary product increasing at a slower rate. Specifically, the evidence suggests that after reaching greater than monthly use for a primary product, youths progress to relatively high levels within 2 years, thus providing insight into how use progresses from experimentation to higher levels.

The 2 trajectories marked by changes in e-cigarette use (increasing e-cigarette/cigarette use and decreasing cigarette/e-cigarette/cigar use) are especially relevant to the current discourse regarding MNPU. The increasing e-cigarette/cigarette use trajectory appears to support the idea that those who initiate nicotine product use with e-cigarettes escalate their use to include other tobacco products,^27^ whereas the decreasing cigarette/e-cigarette/cigar use trajectory may support the use of e-cigarettes for cessation of combustible tobacco use.^28^ Thus, these 2 different classes may be using e-cigarettes for different purposes. To understand these trajectories (and the other trajectories identified), it is important to understand common and unique factors associated with trajectory membership.

We observed that participants in all 5 product use trajectories (ie, classes 2-6 vs the nonusing class) were likely to be in midadolescence, to be male, and to live in homes where others used tobacco and where the use of tobacco was allowed anywhere and anytime. These observations are to be expected given prior research.^6,11,12,13,14,15,16,17,18,19^ As youths become older, they become more likely to try tobacco products and their level of use may escalate.^29^ Thus, effective MNPU prevention strategies are needed to target all stages of adolescence. The sex differences observed are consistent with long-standing evidence of lower rates of tobacco use among girls relative to boys, although the gaps in rates of use have narrowed.^30^ Although boys are at increased risk relative to girls, it is also important for prevention strategies to not overlook girls.^30^ Having a person in the home using tobacco may provide social modeling of this behavior and thus facilitate initiation over time.^29^ Thus, preventive campaigns explaining the impact that tobacco users residing in the home have on youth MNPU need to be tested. Last, although there are strong public health messages on restrictions of combustible product use in private and public spaces,^29^ there is a paucity of compelling public messages regarding the use of noncombustible products in the home. Research on the secondhand impact of noncombustible tobacco products is needed to inform such public health campaigns.

Other socioecological factors were not universally associated with membership in the trajectories. Among the intrapersonal factors, non-Hispanic White participants were more likely to belong to the classes with increasing e-cigarette/cigarette use, stable smokeless tobacco/cigarette use, and decreasing cigarette/e-cigarette/cigar use. These associations are consistent with demographic research showing higher rates of use of tobacco products by White youths.^11^ Given that recent research has indicated cigar use is an important feature of tobacco use among Black youths,^10^ future research should explore whether the increasing cigarette/cigar use class captures these features. In addition, despite the well-established association of low socioeconomic status (SES) with cigarette use,^29^ the decreasing cigarette/e-cigarette/cigar use class was the only trajectory for which there was an association with parental educational level (a proxy for SES). Further, rates of noncombustible product use have been shown to be lower among youths with lower SES relative to the youths with higher SES,^31^ raising uncertainty about the role of SES as a differentiator of future MNPU patterns. Regarding psychiatric symptoms (internalizing or externalizing), reporting symptoms was associated with a greater likelihood of membership in all classes except the stable smokeless tobacco/cigarette use class. It is unclear why an association was not observed for smokeless tobacco use because prior work^32^ has shown an association between smokeless tobacco use and attention-deficit/hyperactivity disorder symptoms. We cannot rule out the possibility that we lacked sufficient power to detect this effect.

Regarding family-level factors, combustible tobacco being allowed (sometimes or anywhere and anytime) inside the home was associated with higher odds of belonging to the increasing cigarette/cigar use, experimentation use, and decreasing cigarette/e-cigarette/cigar use classes. Consistent with the literature,^11^ our findings indicate that youths who reside in homes where combustible product use is allowed are likely to use tobacco products. Although the decreasing cigarette/e-cigarette/cigar use class appears to contradict this association, it should be noted that those in this trajectory reported relatively high levels of use across all 4 waves.

In addition, having a conversation about tobacco use was associated with a higher chance of being in the experimentation, stable smokeless tobacco/cigarette use, and decreasing cigarette/e-cigarette/cigar use classes. It is unclear why this variable would be associated with these classes and not others. Previous research has shown that parent communication about not smoking may contribute to decreased youth smoking,^17,18,19^ so we might expect that experimenters would not escalate their use and cigarette/e-cigarette/cigar users would decrease their use. It is unclear why smokeless tobacco/cigarette use would remain stable. For each of these classes, it may be that parents initiate conversations with these youths because they observe youth tobacco use. Additional exploration is warranted.

At the environmental level, participants who were exposed to tobacco advertising were more likely to belong to all use classes (relative to the nonuse class) except the increasing cigarette/cigar use class. Thus, exposure to tobacco advertising continues to be a risk factor for several patterns of use. The increasing cigarette/cigar use class was the only class that was less likely to report difficulty accessing tobacco products relative to nonusers. This association indicates that an emphasis on reducing accessibility may be especially important for this class.

### Limitations

Our findings should be interpreted within the context of a few study limitations. First, participants were assigned to a class based on most likely fit but may not perfectly follow the observed pattern; therefore, some (maybe less frequent) patterns showing increasing use for each product separately (eg, a class of increasing smokeless tobacco use only) did not emerge. It may be worthwhile for future studies to attempt to replicate these findings. Second, we did not model all the different types of tobacco products used by youths. We could not include hookah use because the use was not measured as the number of days in the past 30 days in the PATH study, and rates of pipe use were too low to model. Third, our model did not include information about the quantity of product use, a common measurement challenge in the tobacco research field. Across products, a uniform metric of quantity of use that can easily be reported is not well established, and measurement of the quantity of e-cigarette and hookah use is especially challenging. Fourth, the PATH study only conducted 1 assessment each year; thus, we could not observe how patterns of use change from month to month. Fifth, the data set did not include data about whether products were used on the same day. Last, because we could not separate days of overlapping use from days of nonoverlapping use, rather than summing days of use to create the combined cigar variable (cigar, cigarillo, and filtered cigar), we created the variable using the maximum days used for a cigar product. This approach may underestimate days of use.

## Conclusions

The findings of this survey study highlight the heterogeneity of longitudinal pathways of MNPU among youths in the US. This study extends previous research because it captured more tobacco products and used a continuous measure of past 30-day use as the outcome. Understanding changes in use patterns and the unique combinations of associated risk factors provides targets for regulatory policies as well as prevention programs directed at youths. Given the limited time that clinicians have during tobacco use screening and counseling, intervention research may find that it is more efficient to provide guidance around the risk factors that are specific to patients’ presenting use patterns.

## Notes

Supplement. **eTable.** Mean Number of Days Each Product Was Used in the Past 30 Days at Each Wave for the Overall Sample and by Class (N = 10 086)
