# Supplementary material for: Longitudinal Trajectories of Multiple Nicotine Product Use Among Youths in the Population Assessment of Tobacco and Health Study
Source: JAMA Netw Open. 2022 Mar 23;5(3):e223549. doi: 10.1001/jamanetworkopen.2022.3549 (PMC8943628; doi:10.1001/jamanetworkopen.2022.3549)
Supplement: Supplement. — eTable. Mean Number of Days Each Product Was Used in the Past 30 Days at Each Wave for the Overall Sample and by Class (N = 10 086) [file jamanetwopen-e223549-s001.pdf]

# Supplementary Online Content

Simon P, Jiang Y, Buta E, Sartor CE, Krishnan-Sarin S, Gueorguieva R. Longitudinal trajectories of multiple nicotine product use among youths in the Population Assessment of Tobacco and Health study. *JAMA Netw Open*. 2022;5(3):e223549. doi:10.1001/jamanetworkopen.2022.3549

**eTable.** Mean Number of Days Each Product Was Used in the Past 30 Days at Each Wave for the Overall Sample and by Class (N = 10 086)

This supplementary material has been provided by the authors to give readers additional information about their work.

© 2022 Simon P et al. *JAMA Network Open*.

| eTable. Mean Number of Days Each Product Was Used in the Past 30 Days at Each Wave for the Overall Sample and by Class (N = 10 086) | | | | | | | |
|---|---|---|---|---|---|---|---|
| | Overall | Non-use (Class 1) | Increasing cigarette/cigar (Class 2) | Experimentation (Class 3) | Increasing e-cigarette/cigarette (Class 4) | Stable smokeless/cigarette (Class 5) | Decreasing cigarette/e-cigarette/cigar (Class 6) |
| Past 30 days use | | | | | | | |
| Cigarette (mean (SD)) | | | | | | | |
| Wave 1 | 0.54 (3.51) | 0.00 (0.04) | 0.21 (0.79) | 0.29 (1.26) | 0.17 (0.63) | 2.31 (6.88) | 14.94 (12.24) |
| Wave 2 | 0.58 (3.46) | 0.00 (0.04) | 1.51 (3.68) | 0.35 (1.28) | 0.80 (2.96) | 2.06 (5.08) | 17.65 (11.33) |
| Wave 3 | 0.67 (3.66) | 0.00 (0.05) | 7.40 (10.00) | 0.43 (1.11) | 1.66 (4.52) | 3.24 (7.32) | 14.13 (12.31) |
| Wave 4 | 0.79 (3.77) | 0.02 (0.15) | 12.10 (11.01) | 0.71 (1.58) | 2.70 (5.26) | 4.09 (7.23) | 10.26 (11.43) |
| Cigar (mean (SD)) | | | | | | | |
| Wave 1 | 0.15 (1.43) | 0.00 (0.02) | 0.36 (2.02) | 0.41 (2.27) | 0.21 (1.25) | 0.43 (1.35) | 2.68 (5.92) |
| Wave 2 | 0.11 (1.12) | 0.00 (0.04) | 0.46 (2.18) | 0.30 (1.20) | 0.28 (1.29) | 1.14 (4.47) | 1.49 (4.28) |
| Wave 3 | 0.16 (1.51) | 0.00 (0.04) | 2.16 (5.94) | 0.23 (0.92) | 0.84 (3.59) | 0.88 (3.60) | 0.81 (3.04) |
| Wave 4 | 0.26 (1.99) | 0.01 (0.12) | 4.77 (8.96) | 0.48 (1.43) | 0.67 (2.27) | 0.60 (2.07) | 0.88 (3.45) |
| E-cigarette (mean (SD)) | | | | | | | |
| Wave 1 | 0.18 (1.75) | 0.00 (0.05) | 0.12 (0.51) | 0.36 (1.80) | 0.97 (4.10) | 0.57 (2.15) | 2.94 (7.40) |
| Wave 2 | 0.37 (2.48) | 0.00 (0.07) | 0.30 (1.11) | 0.43 (1.42) | 4.86 (8.42) | 1.89 (5.88) | 3.18 (7.12) |
| Wave 3 | 0.57 (3.11) | 0.01 (0.14) | 0.63 (2.02) | 0.61 (1.59) | 10.49 (10.45) | 1.57 (4.17) | 2.48 (5.95) |
| Wave 4 | 0.64 (3.24) | 0.03 (0.20) | 1.50 (4.46) | 1.22 (2.28) | 11.27 (11.00) | 1.45 (4.78) | 1.07 (3.39) |
| Smokeless tobacco (mean (SD)) | | | | | | | |
| Wave 1 | 0.18 (2.09) | 0.00 (0.02) | 0.01 (0.09) | 0.07 (0.69) | 0.05 (0.28) | 10.33 (12.96) | 0.28 (1.34) |
| Wave 2 | 0.16 (1.95) | 0.00 (0.03) | 0.01 (0.10) | 0.05 (0.38) | 0.09 (0.71) | 11.02 (12.79) | 0.17 (1.11) |
| Wave 3 | 0.20 (2.07) | 0.00 (0.02) | 0.06 (0.49) | 0.13 (0.78) | 0.37 (2.33) | 13.58 (12.08) | 0.18 (1.09) |
| Wave 4 | 0.19 (1.97) | 0.00 (0.02) | 0.15 (1.09) | 0.26 (1.26) | 0.52 (2.53) | 11.93 (12.48) | 0.14 (0.79) |

© 2022 Simon P et al. *JAMA Network Open*.
